# Endometriotic Follicular Fluid Affects Granulosa Cells’ Morphology and Increases Duplication Rate and Connexin-43 Expression

**DOI:** 10.3390/biom15040561

**Published:** 2025-04-10

**Authors:** Loris Marin, Chiara Sabbadin, Giovanni Faggin, Claudia Maria Radu, Decio Armanini, Michele Paccagnella, Cristiano Salata, Luciana Bordin, Eugenio Ragazzi, Guido Ambrosini, Alessandra Andrisani

**Affiliations:** 1 Department of Women’s and Children’s Health, University of Padova, 35128 Padova, Italy; loris.marin@unipd.it (L.M.); guido.ambrosini@unipd.it (G.A.); alessandra.andrisani@unipd.it (A.A.); 2 Endocrine Unit, Department of Medicine (DIMED), University of Padova, 35128 Padova, Italy; chiara.sabbadin@unipd.it (C.S.);; 3 Department of Molecular Medicine, University of Padua, 35128 Padua, Italy; giovanni.faggin@unipd.it (G.F.); michele.paccagnella.1@studenti.unipd.it (M.P.); cristiano.salata@unipd.it (C.S.); 4 Thrombotic and Haemorrhagic Diseases Unit, Department of Medicine (DIMED), University of Padua, 35128 Padua, Italy; claudiamaria.radu@unipd.it; 5 Studium Patavinum, University of Padova, 35128 Padova, Italy; eugenio.ragazzi@unipd.it

**Keywords:** follicular fluids (FFs), granulosa cells (CGs), endometriosis, connexin-43 (Cx43), follicle-stimulating hormone receptor (FSHR)

## Abstract

Endometriosis is a complicated condition characterized by inflammation, low oocyte quality, and decreased uterus receptivity, associated with fertility issues. This study aims to better understand the reduced pregnancy outcome in endometriosis by analyzing both the granulosa cells (GCs) and the follicular fluids (FFs) obtained during the assisted reproductive technology (ART)-related oocyte pick-up. Seventy patients, approaching our ART Center with the diagnosis of infertility for Age-Idiopathic Factor (AIF) (*n* = 36), endometriosis (ENDO) (*n* = 23), or male factor (MF) (*n* = 11), were enrolled in this study. GCs from each group were separately analyzed for morphology, replication, and expression of Connexin-43 and Follicle-Stimulating Hormone Receptor (FSHR) by microscopy, flow cytometry, and immunocytochemistry. Results show that FF in a culture medium allowed GCs to survive and replicate. Upon culturing GCs from each group with ENDO follicular fluid, increases were observed in both population doublings and in the development of fibroblast-like and muscle-like morphologies. Despite undergoing morphological changes, GCs consistently expressed FSHR. However, exposure to ENDO follicular fluid led to an upregulation of Connexin-43 expression across all GC groups. These findings suggest that in endometriosis, FF contains unidentified factors that can induce aberrant replication, morphological differentiation, and overexpression of Connexin-43, potentially contributing to follicular dysfunction.

## 1. Introduction

Endometriosis (ENDO) is a multifactorial and systemic disease characterized by pain, inflammation, altered pelvic anatomy, adhesions, decreased ovarian reserve, oocyte dysfunction, and compromised endometrial receptivity [1]. Unraveling the mechanisms underlying the association between ENDO and infertility is a challenge whose resolution could lead to the possibility of successfully treating a large number of infertile women. Oocyte quality is one of the most important determinants for successful conception and maternal environment has a key role in the complicated crosstalk between follicular somatic granulosa cells (GCs) and oocytes [2]. Also, inter-communication between GCs is essential for ovarian function, especially for follicle development. GCs communicate directly through gap junctions (GJs), composed of protein subunits called connexins. GJs are not spread uniformly across the entire cell membrane but are concentrated in specific areas where adjacent cells are in contact. These localized clusters, often referred to as “gap junction plaques”, enable efficient and coordinated communication between cells with small molecules, ions, and signaling molecules [2,3]. Through GJs, GCs can share information about hormone levels and metabolic states and regulate oocyte maturation [2]. GJs create contact between GCs and between GCs and the oocyte plasma membrane [4]. The main components are multidomain transmembrane proteins belonging to the family of connexins, in particular Cx43, involved in GC-GC communication, and Cx37, which is involved in GC-oocyte signaling [5].

Follicular fluid (FF), which occupies the antral space of growing ovarian follicles, provides a dynamic microenvironment essential for both follicular development and oocyte maturation, contributing to the acquisition of developmental competence [6]. Bidirectional metabolic and signaling interplay between the oocyte and the surrounding GCs, with the exchange of nutrients, hormones, and signaling molecules through the FF. Alterations in the composition of FF can adversely affect oocyte quality and hinder subsequent embryo development [7].

The inflammation, adhesions, and fibrosis associated with ENDO, which alter the endometrial environment, represent critical determinants of embryo implantation and subsequent development [8,9]. It has been suggested that the altered systemic [10,11] and peritoneal immune and inflammatory profile, characteristic of women with ENDO, directly affects the composition of FF. Altered levels of pro-inflammatory cytokines and growth factors (IL1B, TNFa, IL2, IL8, IL12, IL6, RANTES) have been observed in the FF of women with ENDO compared to healthy controls [12,13,14]. Previous studies have demonstrated spindle and chromosome damage after incubating murine [15] and bovine [16] oocytes in metaphase II with both peritoneal fluid and FF from infertile women with ENDO. Moreover, a significantly reduced implantation rate was evidenced in both rabbits and hamsters following intraperitoneal injection of peritoneal fluid from women with ENDO [17,18].

Research on GC function, especially in humans, has been significantly limited by the inability to culture these cells for extended periods in vitro due to spontaneous luteinization followed by apoptosis occurring within a few days [19].

This study aims to provide deeper insights into the ENDO-related environment that could be involved in potential follicle dysfunction. We compared the effects of FFs on the in vitro development of GCs obtained during assisted reproductive technology (ART) oocyte pick-up in patients with or without ENDO. The results provide new evidence for the presence of factors in FF-ENDO that induce alterations of GCs replications, morphology, and inter-communications which may be consistent with follicle dysfunction. Understanding the complex signaling mechanisms within the follicle and between GCs, the oocyte, and the other tissues in the reproductive system is critical for improving fertility treatments and addressing reproductive disorders.

## 2. Materials and Methods

This is a prospective, single-center study conducted at the In Vitro Fertilization (IVF) Center of the University Hospital of Padua (Italy). Seventy infertile women approaching the IVF treatment were prospectively examined. Informed written consent was obtained from all participants. This study was approved by the Institutional Review Board of the University Hospital of Padua (CET-ACEV 6150/AO/24) and was performed in accordance with the Declaration of Helsinki. The exclusion criteria were as follows: polycystic ovary syndrome, poor ovarian responders defined according to Bologna criteria [20], diabetes, impaired thyroid function, and amenorrhea. Enrolled women were divided into three groups according to their diagnosis of infertility, Age-Idiopathic Factor (AIF), endometriosis (ENDO), and male factor (MF).

### 2.1. Collection of Human Follicular Fluid (FF)

FFs were collected from women undergoing IVF treatment at the Assisted Reproductive Center. Enrolled women underwent controlled ovarian stimulation (COS) with short GnRH antagonist protocol with administration of follitropin-alpha (Ovaleap^®^, Theramex, London, UK) and high purified menotropin (Meriofert^®^, IBSA Institut Biochimique SA, Lugano, Switzerland) from the second or third day of menstrual cycle, with no differences in the total dose of administered gonadotropins and the length of COS. The FSH dose was given according to ovarian reserve parameters (antral follicle count and AMH level). All subjects received daily subcutaneous injections of 0.25 mg ganirelix (Orgalutran^®^, Organon, Amsterdam, The Netherlands) when at least one follicle diameter was 14 mm until the day of Human Chorionic Gonadotropin (hCG) administration. A total of 250 mcg of Recombinant Human Chorionic Gonadotropin (rhCG) (Ovitrelle^®^, Merck Serono S.p.A., Darmstadt, Germany) was administered when the largest cohort of follicles was greater than 16 mm and 36 h later follicle aspiration was carried out. During oocyte retrieval, follicular fluid was collected from all aspirated follicles. Small follicles (<14 mm) were left unpunctured according to European Society of Human Reproduction and Embryology (ESHRE) guidelines [21].

The FF was obtained during the transvaginal oocyte retrieval. The cellular content of the aspirated FF consisted of a mixture of luteinizing GCs, both single and in clumps, erythrocytes, and large epithelial cells. For each patient, the FF from each follicle was pooled excluding that from the first tube to avoid the contamination of vaginal epithelial cells.

The collected FF was immediately centrifuged at 200× *g* for 10 min to separate GCs and collect them. The supernatant was further centrifuged at 4500× *g* for 10 min and stored at −20 °C.

Once thawed, FF was centrifuged again at 4500× *g* for 10 min and the supernatant was sterilely filtered with a 0.22 mm pore size filter and stored at −20 °C till the use.

The variability of FF characteristics was overcome by pooling together the FF of 2–3 women with the same infertility diagnosis (AIF, ENDO, MF). Potential additional bias within each group was minimized by selecting samples from patients with the greatest age differences and varying ENDO stages. Additionally, the pooling of FF was conducted independently of granulosa cell (GC) pooling to minimize the influence of patient-specific factors. This approach ensured that any variations observed in pooled GCs were not directly mirrored by a specific patient’s FF composition, reducing the risk of patient-related biases affecting the analysis.

### 2.2. GC Cultures

GCs and FF were each divided into three groups based on women’s infertility diagnosis. Collected cells were washed twice by centrifugation at 200× *g* for 10 min at room temperature with culture medium (CM), consisting of Dulbecco’s Modified Eagle’s Medium (DMEM/F12 Thermo Fisher Scientific, Waltham, MA, USA), 8% fetal bovine serum FBS (Thermo Fisher Scientific), 10,000 units/mL penicillin, and 10,000 μg/mL streptomycin (Thermo Fisher Scientific). Cells were cultured at 37 °C under aerobic conditions (5% CO_2_) for 24–36 h to let the alive GCs adhere. Cultures were successively washed in CM to remove the apoptotic and contaminant cells and the CM was substituted with fresh ones.

After 7 days of culture, adherent cells were treated with TrypleExpress (Thermo Fisher Scientific) for 7 min at 37 °C according to the manufacturer’s instructions and counted using a Makler counting chamber (SEFI Medical Instruments Ltd., Haifa, Israel). A total of 5 × 10^4^ and 5 × 10^6^ cells were seeded in 12-well plates with 1 mL CM or in T25 Flask with 5 mL CM, respectively, with or without the addition of 20% FF from different groups. Cultures were maintained at 37 °C in a humidified atmosphere containing 5% CO_2_. Non-adherent cells were discarded after 48 h, and the medium was replaced twice a week.

To overcome the variability related to individual characteristics, GCs from 2 to 3 women belonging to the same group were pooled. Potential additional bias within each group was minimized by selecting samples from patients with the greatest age differences and varying ENDO stages and taking care to avoid GCs being treated in the presence of the corresponding FF.

### 2.3. Population Duplication Count

Cells were dissociated using the recombinant protease TrypLEExpress (Gibco™, by Thermo Fisher Scientific Inc., Waltham, MA, USA) according to the manufacturer’s instructions. After cell detachment, the enzymatic activity was stopped by dilution with CM. Cells were centrifuged for 5 min at room temperature at 750× *g*. The cell pellet was resuspended in CM and evaluated for viability with trypan blue exclusion, and viable cells were counted.

For the population doubling (PD) assay, 5 × 10^4^ cells were seeded in a 12-well plate and cultured at 37 °C for 21 days. On the 7th, 14^th^, and 21st day, cells were harvested and counted as above described.

The number of PDs achieved with the culture was calculated at each passage with the formula:

*PDs* = (*logN_T_* − *logNo*)/*log*2

where *N_T_* is the number of cells recovered during harvesting and *No* is the number of cells originally plated.

### 2.4. GCs Morphology Evaluation

Phase-contrast microscopic images were obtained by a Nikon Eclipse TE2000S Microscope (Nikon Corporation, Tokyo, Japan) equipped with a Cooper Surgical DC1 camera.

Images of differently incubated GCs (10 fields for at least 200 cells) were taken every 3 days and evaluated with CellProfiler (https://cellprofiler.org/), an open-source software to quantitatively measure cell phenotypes from thousands of images by two separate expert biologists during all incubation process [22]. Data were collected and statistically analyzed.

### 2.5. Flow Cytometry Analysis

Cells were seeded into 12-well plates and cultured at 37 °C for 7 days in the presence of different media, then collected in centrifuge tubes and washed three times with PBS.

For apoptosis assay, cells were stained with FITC Annexin V Apoptosis Detection Kit I (BD Biosciences, Franklin Lakes, NJ, USA) according to the manufacturer’s instructions.

Intracytoplasmic staining of Connexin-43 (Cx43) and follicle-stimulating hormone receptors (FSHR) were performed using FIX and PERM (Invitrogen, by Thermo Fisher Scientific, Waltham, MA, USA) according to the manufacturer’s instructions. The samples were incubated with the primary antibodies for 45 min at room temperature: mouse-monoclonal anti-Cx43 (Invitrogen, by Thermo Fisher Scientific, Waltham, MA, USA) and polyclonal rabbit anti-human IgG FSHR (Invitrogen, by Thermo Fisher Scientific, Waltham, MA, USA). After washing, samples were stained with Alexa Fluor™ 488 Goat anti-Mouse IgG (H + L, Invitrogen, Waltham, MA, USA) and Alexa Fluor™ 594 Goat anti-Rabbit IgG (H + L, Invitrogen, Waltham, MA, USA) secondary antibodies and resuspended in PBS supplemented with bovine serum albumin (BSA) 5%. The acquisition was performed with a BD LSR-II cytometer (BD Biosciences, Franklin Lakes, NJ, USA) and analyzed by FlowJo software (version 10.8.1, TreeStar Inc., San Carlos, CA, USA). Quantitative analysis for FSHR and CX43 expression was performed by considering the difference between Mean Fluorescence Intensity (MFI) values and MFI autofluorescence control values (cells stained with secondary antibody only).

### 2.6. Immunocytochemistry (ICC)

GCs were seeded onto small glass coverslips placed into 12-well plates and allowed to grow in different media for 7 days (up to 60–70% confluent). The coverslips were removed, washed with PBS three times, fixed with FIX and PERM™ Cell Permeabilization Reagents (Invitrogen, by Thermo Fisher Scientific, Waltham, MA, USA) for 15 min at room temperature according to the manufacturer’s instructions, washed with PBS three times, and blocked for 30 min with FBS 3% in PBS at RT. The samples were treated at 4 °C with the primary antibodies, mouse-monoclonal anti-CX43, and polyclonal rabbit anti-human IgG FSHR followed by stain with goat anti-Rabbit IgG (H + L, Invitrogen) (H + L, by Invitrogen) Alexa Fluor™ 594 or and goat anti-Mouse IgG (H + L, Invitrogen, Waltham, MA, USA) Alexa Fluor™ 488 secondary (H + L, by Invitrogen) antibodies, respectively, at room temperature for 30 min. Samples were rinsed with PBS before acquisition. The cell nuclei were stained with DRAQ5™ Fluorescent Probe (Thermo Fisher Scientific) for 25 min at room temperature. Images were acquired with a Nikon A1RSi Laser Scanning inverted confocal microscope (Nikon Corporation, Tokyo, Japan) equipped with NIS-Elements Advanced Research software (Nikon Instruments Inc., Melville, NY, USA).

### 2.7. Statistical Analysis

The statistical analysis was performed using JASP computer software (Version 0.18.3, JASP Team, Amsterdam, The Netherlands, 2024) working with R language (Version 4.3, R Core Team, Vienna, Austria, 2023). Continuous data are expressed as means ± SD. Statistical significance was obtained with one-way ANOVA followed by Tukey’s post hoc test. Frequency data are expressed as absolute values or percentages; statistical significance was assessed by means of a chi-squared test followed by Fisher’s exact approach [23].

## 3. Results

### 3.1. Patients’ Characteristics

Seventy patients presenting at our ART Center with a diagnosis of infertility were enrolled in this study and their FF was processed.

There was a significant difference in the age in the groups, with the mean age of women in group AIF being 38.06 ± 3.13 y, 35.30 ± 3.23 y in ENDO, and 33.64 ± 1.21 y in group MF (*p*-value < 0.001 and <0.0001, respectively).

There was not a significant difference in body mass index (BMI) (22.56 ± 3.2, 23.49 ± 3.51, 23.43 ± 4.27 in group AIF, ENDO and MF, respectively) or in the antral follicle count (AFC) (10.36 ± 5.5, 9.57 ± 6.06, 12.83 ± 4.84) and Anti-Müllerian Hormone (AMH) (1.9 ± 0.71, 2.08 ± 1.38, and 2.43 ± 1.25, respectively). The follicle output rate (FORT), defined as pre-ovulatory follicle count on the day of ovulation trigger, and Follicle-to-Oocyte Index (FOI), defined as the rate between the number of oocytes retrieved at oocyte pick-up and the number of antral follicles before stimulation, showed no significant differences.

Patients’ characteristics are summarized in Table 1.

**Figure 1:**
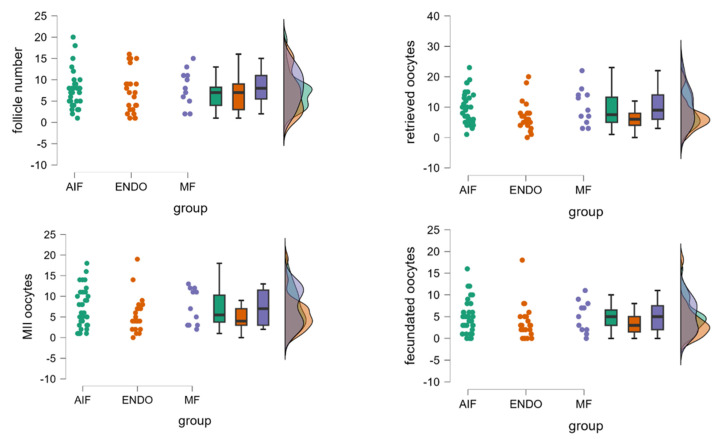
Comparison of follicle number and oocyte quantity and quality among MF, AIF, and ENDO groups. The panels represent the number of follicles, number of retrieved oocytes, number of mature oocytes (metaphase II), and number of fecundated oocytes, evaluated in the three groups. Each raincloud plot shows raw data points, box plots (displaying median and interquartile range), and a violin plot (showing the kernel density estimate), thereby providing a comprehensive view of the data distribution, central tendency, and variability. Each color corresponds to one of the three specific groups, with the same color scheme consistently applied to the raw data points, box plots, and violin plots to maintain visual coherence. The density plot suggests a high degree of superimposition among the three groups, indicating overlapping distributions. No significant differences among the three groups (AIF, ENDO, and MF) were observed, using one-way ANOVA.

There was not a significant difference in the number of follicles ≥ 14 mm during COS, in the number of retrieved oocytes, and in the number of MII oocytes. Details of ovarian stimulation outcomes are reported in Table 2 and Figure 1. When analyzed for the pregnancy rate, there were differences among the groups, being more than double in MF and AIF compared to ENDO (29% and 24% compared to 11%, respectively); however, these differences did not reach statistical significance according to the chi-square test (*p* = 0.483), possibly due to the limited sample size in this pilot investigation, which may have affected the power to detect a significant difference for this outcome.

### 3.2. Morphological Classification of Cells Recovered from Pick-Up

GCs were classified into three sub-populations: follicle epithelial-like GCs (FELGC), fibroblast-like GCs (FLGCs), and undifferentiated GCs (uGCs) [25].

**Figure 2:**
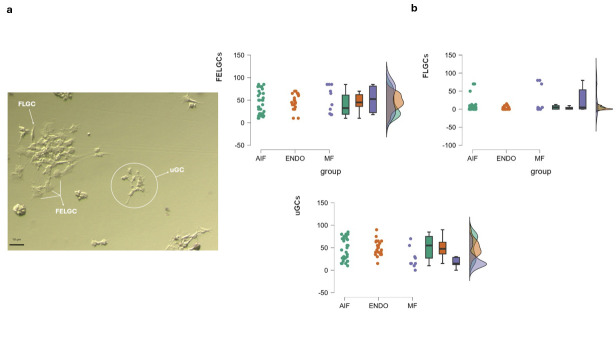
GCs sub-populations. (**a**) Morphological features of GC sub-populations. Cells collected at the time of pick-up were cultured for 24–36 h, followed by washing in CM to remove apoptotic cells. They were then incubated in CM for an additional 4–5 days to allow for stabilization and development before undergoing morphological evaluation, as detailed in Section 2 Morphological features of GC sub-populations retrieved at pick-up. FELGCs: follicle epithelial-like granulosa cells; FLGCs: fibroblast-like granulosa cells; uGCs: undifferentiated granulosa cells. (**b**) Raincloud plots showing morphological distribution of three GC sub-populations at pick up inside the three groups. The color codes are the same as those used in Figure 1 for consistency. No significant differences among the three groups (AIF, ENDO, and MF) were observed for each GC sub-population, using one-way ANOVA. The density plot suggests a high degree of superimposition among the three groups, indicating overlapping distributions.

FELGCs appeared as large flat rough cells with no filaments/protrusions. They easily adhered to either the plate or flask but were unable to re-adhere after passage. FLGCs were characterized by a long and tapered shape similar to fibroblasts and were characterized by long extensions seeking contact with other cells. FLGCs easily survived and re-adhered once passaged. uGCs were characterized by small irregular bean-shaped cells, surrounded by multiple extrusions, and were able to survive and re-adhere after passage (Figure 2).

GC morphology, evaluated immediately after the oocyte retrieval, showed GC sub-populations in all groups. FELGCs were similarly distributed in the three groups, while uGC and FLCG distribution was significantly different among women with different infertility diagnoses. In particular, there were significantly fewer uGCs in the MF group (23.81 ± 21%) than in the AIF and ENDO groups (51.16 ± 25.37%, *p* = 0.002, and 49.45 ± 17.02% *p* < 0.008, respectively). FLCGs were higher in the MF group, (24.8 ± 35.95%) than AIF (9.16 ± 17.25%), although this difference did not reach statistical significance (*p* = 0.056); instead, FLCGs were significantly higher in the MF group compared to the ENDO group (3.68 ± 4.57%, *p* = 0.011).

### 3.3. Effect of FF on GC Survival

To assess if the different sub-population ratios characterizing the GCs would affect their in vitro survival, GCs were incubated with CM alone and with CM supplemented with FF-AIF, FF-ENDO, and FF-MF.

The results showed that CM alone was not able to support cell survival and replication and led cells to apoptosis (apoptotic cells 70.05 ± 16.69%, *p* < 0.05). Conversely, the apoptotic process was nearly absent in the GCs from all groups when incubated with FF-supplemented CM, irrespective of both the specific GC group and the FF origin. This suggests a protective or survival-promoting effect of FF on granulosa cells, potentially supporting their viability and function.

**Figure 3:**
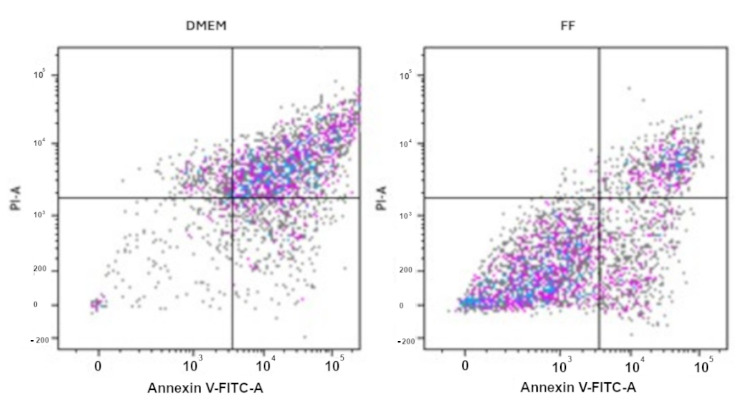
Annexin V-PI Apoptosis essay on GCs. GCs were cultured in the absence (CM) or presence of FF from AIF, ENDO, or MF patients for 14 days, collected, stained with Annexin V/PI, and measured by flow cytometry, as described in Materials and Methods. The panel of FF represents all the different treatments (FF-AIF, FF-ENDO, FF-MF). The figure is representative of 3 separate experiments for each group of GCs. Coloured dots represent cells, in particular the **Lower Left Panel (Annexin V−, PI−)** represents **viable cells**, or cells negative for both stains and not undergoing apoptosis or necrosis; the **Lower Right Panel (Annexin V+, PI−)** represents **Early apoptotic cells**, positive for Annexin V but negative for PI, presenting externalized, phosphatidyl serine (PS) but still intact membrane; the **Upper Right Panel (Annexin V+, PI+)** for **Late apoptotic or necrotic cells** with PS externalization and compromised membrane; the **Upper Left Panel (Annexin V−, PI+)** for the **Necrotic cells** with mechanical damage, compromised membrane but withoue PS exposure, i.e., negative for Annexin V, positive for PI.

We did not observe any difference among FF-AIF, FF-ENDO, and FF-MF groups (apoptotic cells 39.99 ± 3.66%, 36.67 ± 6.00%, and 34.37 ± 3.96%, respectively (Figure 3).

### 3.4. Effect of FF on GCs Morphological Differentiation

GCs were incubated in the presence of corresponding FF-CM for up to 3 weeks, and their morphology was reported. Notably, FELGC and uGC sub-populations disappeared in all the different groups of GCs. In addition, four new morphological sub-populations were observed (Figure S1): epithelial-like GCs (ELGCs) with the classic cobblestone morphology, forming tightly packed colonies with well-defined cell junctions [25,26], indicative of strong intercellular adhesion, potentially suggesting a role in barrier formation and cell-to-cell communication; chondroblast-like GCs (CLGCs), as disc-shaped cells surrounded by a smooth extracellular matrix, resembling early chondroblasts involved in cartilage formation. Their morphology suggests active matrix deposition and potential involvement in extracellular matrix remodeling, contributing to tissue structural integrity. The third sub-population was represented by the muscle-like GCs (MLGCs), as spindle-shaped cells with broad central regions and tapering ends, resembling myoblasts or fibroblast-like cells. Their elongated structure may imply a capacity for contractility and mechanical support, potentially playing a role in fiber tissue stiffness and repair mechanisms. The last sub-population, the neuronal-like GCs (NLGCs), had an elongated morphology and extended processes, and resembled neural precursor cells. Their shape suggests potential roles in cell signaling, network formation, or responsiveness to neurogenic cues, indicating a possible involvement in neuroendocrine-like functions.

**Figure 4:**
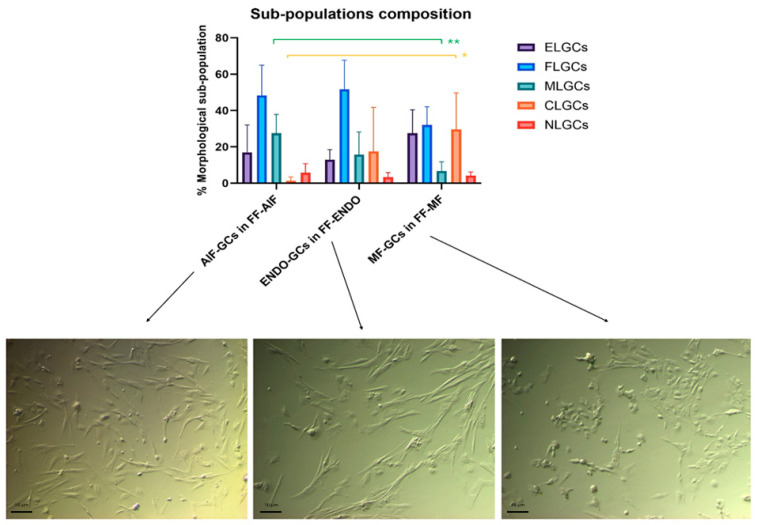
Comparison among the sub-population components in the three groups incubated in CM added with the corresponding FF. GCs from AIF (AIF-GCs), ENDO (ENDO-GCs) or MF (MF-GCs) were incubated in CM added with FF from AIF (FF-AIF), ENDO (FF-ENDO) or MF (FF-MF) for 21 days. Morphology was evaluated by two different expert biologists by analyzing 10 different fields/plates. The number of the different morphological cells was reported and represented as a percentage. A total of 200 cells/samples were evaluated and expressed as percentage mean value ± SD. The figure is representative of six separate experiments for each group. ELGCs: epithelial-like granulosa cells; FLGCs: fibroblast-like granulosa cells; MLGCs: muscle-like granulosa cells; CLGCs: chondroblast-like granulosa cells; NLGCs: neuronal-like granulosa cells. Statistical differences were assessed by comparing for the three groups the effects of the three FF treatments (FF-AIF, FF-ENDO, and FF-MF) on GC morphological differentiation (ELG, FL, ML, CL, and NL). * *p* = 0.042 and ** *p* = 0.006 indicate statistical significance based on Tukey’s post hoc test after ANOVA, comparing “AIF-GCs in FF-AIF” vs. “MF-GCs in FF-MF”. Scale bar = 10 μm.

FLGC was the largest sub-population in all groups. Specifically, it represented 48.33 ± 16.63% in AIF-GCs, 51.67 ± 16.02% in ENDO-GCs, and 32.00 ± 10.10% in MF-GC groups (Figure 4).

When GCs were treated with the corresponding FF, there was a significantly different sub-population composition among women belonging to different groups.

In particular, in the MF-GCs group, the CLGCs were significantly higher (29.67 ± 20.12%, being almost one-third of the total MF-GCs, vs. 1.33 ± 2.16 %in AIF-GCs, *p* = 0.042) and MLGC significantly lower than AIF-GCs (6.67 ± 5.16% in MF-GCs vs. 27.50 ± 10.37% in AIF-GCs, *p* = 0.006).

**Figure 5:**
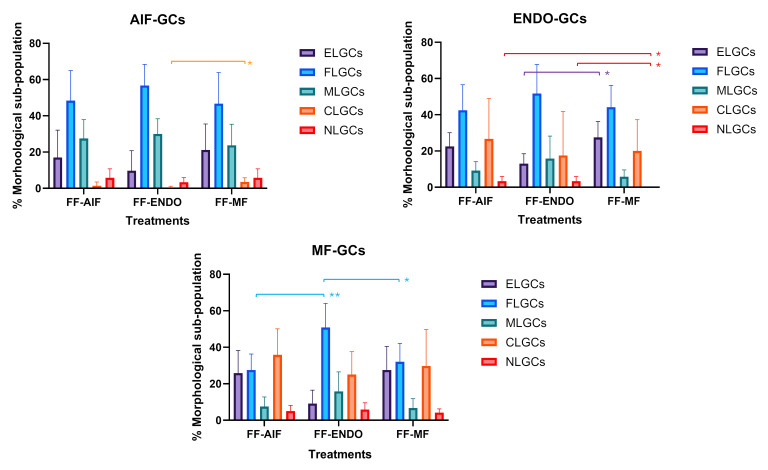
Effect of different treatments on GCs morphological differentiation. GCs from AIF (AIF-Group) ENDO (ENDO-Group) or MF (MF-Group) were incubated in CM added with FF from AIF (FF-AIF), ENDO (FF-ENDO) or MF (FF-MF) for 14 days. Morphology was evaluated by two different expert biologists by analyzing 10 different fields/plates and the number of the different morphological cells reported and represented as a percentage. A total of 500 cells/samples were evaluated and expressed as percentage mean value ± SD. The figure is representative of six separate experiments for each group. ELGCs: epithelial-like granulosa cells; FLGCs: fibroblast-like granulosa cells; MLGCs: muscle-like granulosa cells; CLGCs: chondroblast-like granulosa cells; NLGCs: neuronal-like granulosa cells. Within the three GC groups (AIF-GCs, ENDO-GCs, and MF-GCs), statistical differences were assessed by comparing the effects of the three FF treatments (FF-AIF, FF-ENDO, and FF-MF) on GC morphological differentiation (ELG, FL, ML, CL, and NL). * *p* < 0.05 and ** *p* < 0.01 indicate statistical significance based on Tukey’s post hoc test after ANOVA.

When GCs were cultured with the FF of different groups, CG morphology alteration was more evident (Figure 5).

A significant increase in the CL-GC sub-population was observed in AIF-GCs in the presence of FF-MF compared to FF-ENDO.

In ENDO-GCs, the presence of FF-MF significantly increased the EL-GCs and NL-GCs sub-populations and showed a tendency to reduce ML-GCs. In MF-GCs, the effect of FF-ENDO led to a significant increase in the FL-GC sub-population.

In all GCs, FF-AIF induced changes similar to, but less pronounced than, those observed in cultures with FF-ENDO.

### 3.5. GC Population Duplications (PDs)

**Figure 6:**
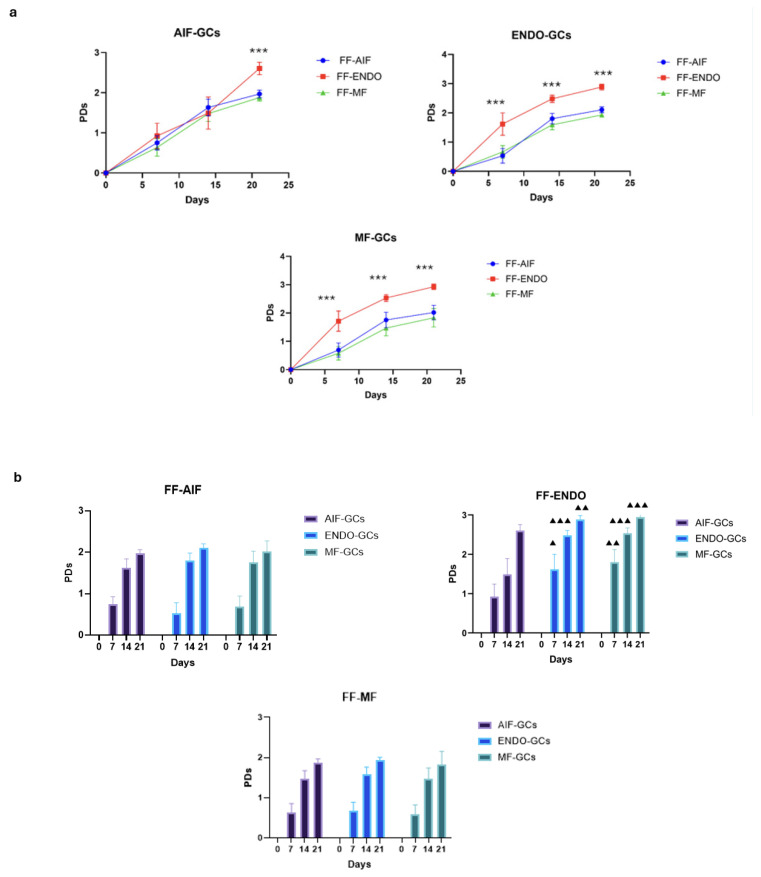
Population replications. Cells were seeded in a 12-well plate, incubated in the presence of CM added with FF-AIF, FF-ENDO, or FF-MF, and let grow. After 7, 14, and 21 days, the cells were harvested and counted as above described. The number of population doublings (PDs) is calculated from the formula PDs = (logNT − logNo)/log2, where NT is the number of cells recovered during harvesting at time T, and No is the number of cells originally plated. Values are expressed as mean ± SD of six separate experiments. (**a**) Graphical representation of the PDs curve obtained by GCs belonging to each group.in the presence of different FF-CM. *** *p* < 0.0001, Tukey’s post hoc test, comparison FF-ENDO vs. FF-AIF and FF-MF at respective times. (**b**) Graphical representation of the PD curve obtained by each FF-CM on the three different groups of GCs. ▲ *p* < 0.05; ▲▲ *p* < 0.01, ▲▲▲ *p* < 0.001, Tukey’s post hoc test, comparison AIF-GCs vs. ENDO-GCs, and AIF-GCs vs. MF-GCs, at respective times.

The PD curves of the three GC groups cultivated in the presence of FF-CM are shown in Figure 6a. The increase in PDs was observed in all cell cultures (reaching almost 3 PD at 21 days) and the increase induced by FF-ENDO was significantly greater than that of the other two FF (*p* < 0.0001) for all the three GCs groups. Also, FF-ENDO significantly increased the rate of PDs reaching higher values already after 7 and 14 days both in ENDO-GCs (*p* < 0.05 at 7 days; *p* < 0.001 at 14 days, compared to AIF-GCs) and in MF-GCs (*p* < 0.01 at 7 days; *p* < 0.001 at 14 days, compared to AIF-GCs) (Figure 6b). Interestingly, both FF-AIF and FF-MF were able to reduce the replication rate of ENDO-GCs (Figure 6b).

### 3.6. GCs FSHR and Connexin-43 (Cx43) Evaluation

To assess the identity of the cultured cells as granulosa after incubation, GCs were analyzed by the presence of FSH receptor (FSHR) [13]. No changes in FSHR expression were present in the cultured GCs in any conditions (Figure S2).

**Figure 7:**
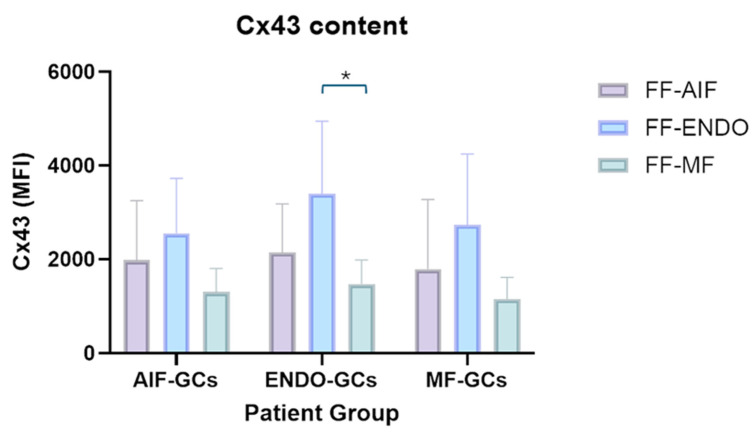
Effect of FF-AIF, FF-ENDO, and FF-MF on AIF-GCs, ENDO-GCs, and MF-GCs- Cx43 content. GCs, recovered as described in Materials and Methods, were incubated in the presence of CM supplemented with FF from each patient group for 21 days. Cells were detached, washed, fixed, permeabilized, and labeled with anti-Cx43 antibody, following customer guidelines. Fluorescence was recorded by flow cytometry and expressed as MFI mean values ± SD of six separate experiments. * *p* < 0.05, Tukey’s post hoc test.

**Figure 8:**
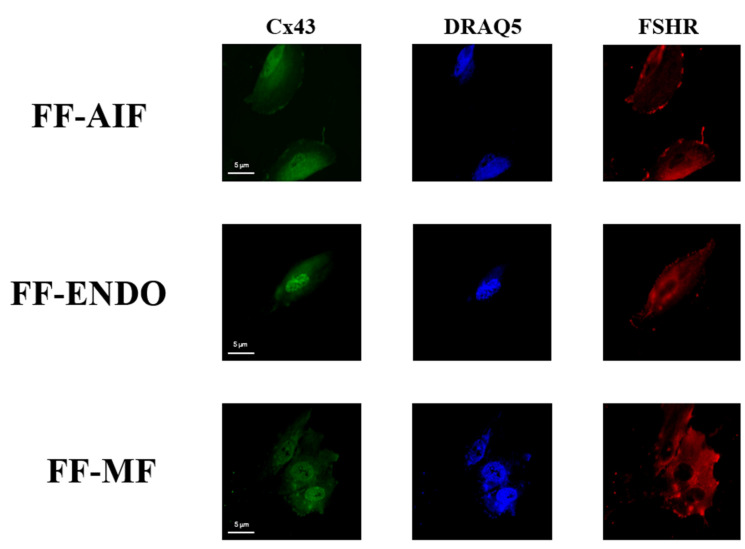
Cx43 and FSHR distribution in GCs under different conditions. Representative immunofluorescent images of GCs incubate with CM containing FF-AIF, FF-ENDO, or FF-MF for CX43 and FSHR cellular distribution. Images of GCs were analyzed by confocal microscopy to visualize FSHR (polyclonal rabbit anti-FSHR with Alexa Fluor 594) and Cx43 (monoclonal mouse anti-Cx43 with anti-Alexa Fluor 488) The figure is representative of 3 different experiments for each condition. Scale bar = 5 μm.

The effects of the three different treatments (FF-AIF, FF-ENDO, and FF-MF) were also investigated on the ability of GCs to express Cx43 (Figure 7 and Figure 8).

In the three GC groups, treatment with FF-ENDO determined an increase in the expression of Cx43 compared to the effect of FF-MF, with the highest level of Cx43 in ENDO-GCs. Treatment with FF-MF caused a decrease in Cx43 content; notably, Cx43 expression in ENDO-GCs was comparable to those of MF-GCs and AIF-GCs in such conditions. FF-AIF induced Cx43 expression higher than FF-MF but lower compared to FF-ENDO in all three groups of GCs.

In Figure 8, the subcellular localizations of both Cx43 and FSHR are reported. The scattered red fluorescence described a diffuse presence of FSHR in all three differently supplemented conditions, whereas the green fluorescence suggested a perinuclear localization of Cx43 in the presence of FF-ENDO, compared to both other treatments.

## 4. Discussion

GC morphological changes and survival have traditionally been observed in studies involving specific treatments, such as exposure to specific neuroinductive, chondroinductive, and osteoinductive culture media [25,27,28] or transfection with RNA/DNA vectors [29].

In this study, we analyzed the impact of FF from three distinct groups (AIF, ENDO, MF) on GC morphology and differentiation. Our findings revealed the spontaneous emergence of four distinct GC sub-populations—fibroblast-like, muscle-like, epithelial-like, and chondroblast-like cells—in the absence of exogenous differentiation inducers. This highlights the intrinsic capacity of FF not only to support GC survival and proliferation but also to actively modulate their differentiation. Notably, the composition of FF, particularly in the context of endometriosis, preferentially promotes the differentiation of GCs into fibroblast- and muscle-like phenotypes. These results suggest that FF acts as a key pathology-related microenvironmental factor influencing the terminal differentiation of GCs.

Despite the recognized association between ENDO and infertility, the multifactorial nature of the disease significantly complicates the understanding of the underlying mechanisms involved. In ART procedures, the quality of gametes remains a primary concern, but it represents only one of the limiting factors in achieving successful pregnancy and live birth. Another crucial factor is the maternal environment, which not only plays a role in the development of high-quality gametes through the complex interactions between follicular somatic cells (GCs) and oocytes but also provides the optimal conditions necessary for embryo implantation [30,31].

During follicle development, GC proliferation is sustained by hormonal activity until ovulation when the Luteinizing Hormone (LH) peak (or its ART homologous hCG) acts on the ovary inducing the rupture of the dominant follicle. After ovulation GCs start luteinizing with marked functional changes [32]. In physiological conditions, this sequence of multiple molecular and phenotypical rearrangement drives GCs into the formation of Corpus Luteum (CL) for supporting the successive embryo implant through progesterone secretion. In the absence of conception, CL is subjected to a quick regression with the transformation into connective tissue. The luteinized GCs are thus destined for a short life expectancy and this prevents them from being cultured long-term in vitro [33]. It has been previously assessed that GCs progressively lose their ability to express FSHR towards a sort of spontaneous luteinization during in vitro cell culture, changing their own morphology and function [34]. In our experiments, when cultured in the presence of FF from AIF, ENDO, or MF groups, GCs showed no changes in their FSHR expression. The ability of FF to support GCs’ long-term survival in in vitro cultures has been previously evaluated [28]. Horisberger [33] assured that even after 45 days, GCs maintained their granulosa main characteristic, represented by the expression of FSHR. This observation suggests that under these experimental conditions, FSHR expression does not fluctuate with prolonged culture time, passaging, or morphological alterations, whereas the absence of FF, regardless of the patient group, did not allow cell survival and replication and led cells to apoptosis.

The different GCs morphology, GCs population duplication, and Cx43 expression under different culture conditions can help to understand how the environment of certain pathological environments can affect GCs and consequently lead to follicle dysfunction. FF comprises a plethora of factors including different-sized extracellular vesicles (EVs), ions, vitamins, and hormones. Assuming that all the recruited patients were subjected to the same COS protocol for ART treatment, differences in GC behavior with different FF should rely on the corresponding pathophysiological conditions. We demonstrated that ENDO-FF induced GCs to preferred fibroblast-like or muscle-like morphologies, regardless of their belonging group (AIF, ENDO, or MF). It is known that one result of ENDO is fibrosis, which is characterized as overly dense fibrous tissue around endometrial glands and stroma [35,36]. To the best of our knowledge, this is the first study comparing the effects of FF in women with different infertility diagnoses (MF, AIF, and ENDO) on GCs.

Many FF components have been linked to aberrant signaling in ENDO including plasma-derived osmotic fluid, steroids, metabolites, polysaccharides, proteins, peptides, reactive oxygen species (ROS), antioxidant enzymes, and EVs [37]. It has been reported that in ENDO peritoneal fluid EVs carry molecules modulating immune response and promoting the establishment and maintenance of endometriotic lesions [37]. EVs are produced by and carry a wide range of different components in response to stress or physiological changes in the tissue’s homeostasis. Recently, growing interest has been devoted to the formation of these intercellular mediators which are released into the extracellular environment of many body compartments and serve as vehicles for the transfer of proteins, lipids, and RNAs between cells both locally (autocrine and paracrine) and remotely [38]. In animal models, it has been shown that EVs have the capacity to be internalized by oocytes, cumulus cells, and GCs [39].

The EVs’ property of carrying short RNA sequences made them recognized as important factors regulating the epithelial-mesenchyme transition (EMT), a biological process that converts normal polarized, cobble-stone-epithelial-like cells into fibroblastic (elongated) mesenchyme cells, with the consequence of altering structural epithelial integrity and functions. In both cases, the FF-ENDO EVs might be responsible for the differentiation processes that have been shown in the long-term culture of the GCs from all three groups (AIF, ENDO, and MF). In addition, EVs derived from ENDO FF may carry specific lncRNAs able to influence GC behavior by regulating gene expression, modulating transcription, or altering signaling pathways involved in GC differentiation, proliferation, and function. In endometriosis, altered EV cargo may contribute to the increased presence of muscle-like and fibroblast-like phenotypes thus promoting an inflammatory or fibrotic environment, disrupting normal luteinization, and impairing Cx43-mediated communication. This EV-mediated transfer of lncRNAs could therefore play a key role in shaping GC sub-populations and ovarian dysfunction in women with endometriosis.

However, the predominant mode of communication between GGs and between GGs and oocytes is mediated through GJs, which facilitate direct intercellular exchange of ions, small molecules, and signaling molecules critical for oocyte development and follicular dynamics. Cx43 is a key protein involved in the formation of GJs, which are specialized intercellular channels that allow for direct communication between adjacent cells. In the context of the ovarian follicle, Cx43 plays a crucial role in communication between GCs and between GCs and oocytes, which is essential for proper oocyte development and maturation [40].

While increased Cx43 expression might suggest enhanced intercellular communication, this is not necessarily the case, as its functional activity is tightly regulated by phosphorylation. Specifically, phosphorylation at tyrosine residues has been associated with an inactivated state of Cx43 [41], meaning that increased expression could instead reflect a compensatory response to maintain gap junction function. Moreover, the activation of Cx43 requires dephosphorylation, a process mediated by P-Tyr phosphatases. However, these enzymes are known to be inhibited by oxidative stress [42], which is a hallmark of endometriosis [10,11]. As a result, even with increased Cx43 expression, oxidative stress-induced phosphatase inhibition may lead to persistent Cx43 inactivation, further impairing granulosa cell communication and follicular function.

In addition, increased CCx43 expression can also interfere with the delicate regulation needed for proper oocyte and follicle growth and maturation, leading to follicle dysfunction with reduced oocyte quality and impaired fertilization potential in women with ENDO. Moreover, the internalization and altered localization of Cx43 could impair cellular responses to extracellular signals, including those involved in oocyte meiotic regulation. This mislocalization of Cx43 may disturb the delicate balance of signaling pathways, further compromising the intricate processes necessary for the successive correct luteinization

Enhanced motility, migratory-invasive ability, higher resistance to senescence and apoptosis, together with increased production of Extra Cellular Matrix (ECM) components are the main features of mesenchymal cells [43,44]. EMT represents an important phase in development, differentiation, inflammation, fibrogenesis, and tumorigenesis [45].

The observed muscle-like and fibroblast-like morphologies in granulosa cells may indicate a predisposition to dysregulated luteinization. Such phenotypic alterations can impair the essential tissue and ECM remodeling required for proper corpus luteum formation, a process vital for progesterone production and early pregnancy support. Effective ECM remodeling involves precise synthesis and degradation of matrix components, crucial for follicular development and luteal function.

Furthermore, the potential impairment of Cx43 communication during this phase could disrupt the coordinated signaling necessary for proper luteal cell function and progesterone secretion, further supporting the idea that these morphological changes reflect an altered luteal phase rather than an issue with oocyte maturation.

Further studies are needed to elucidate the precise mechanisms by which Cx43 expression and localization influence GC communication, oocyte maturation, and follicular development in ENDO. A deeper understanding of these processes may pave the way for targeted therapeutic strategies to improve fertility outcomes in affected women.

## 5. Conclusions

FF plays a pivotal role in the development of both the follicle and the oocyte, facilitating bidirectional communication that nourishes GCs, which in turn support oocyte maturation. Our findings indicate that ENDO predominantly affects the composition of FF. Notably, while GCs from patients with ENDO exhibit similar morphological characteristics, replication rates, and Cx43 protein expression levels as GCs from non-ENDO individuals (AIF-GCs and MF-GCs), exposure to FF-ENDO leads to a selective increase in MLGC and FLGC morphologies, along with enhanced replication and elevated Cx43 protein expression. These alterations may serve as significant targets for therapeutic interventions aimed at addressing infertility in patients with ENDO.

## Figures and Tables

**Table 1:** Patients’ parameters. Total FSH dose (expressed as international unit) administered during COS to each patient, Body Mass Index (BMI), and antral follicle count (AFC) were calculated for the evaluation of the ovarian sensitivity; Anti-Müllerian hormone (AMH). Follicle output rate (FORT) > 0.30 as normal ovarian sensitivity, defined as pre-ovulatory follicle count on the day of ovulation trigger; Follicle-to-Oocyte Index (FOI) > 0.50 for normal number of oocytes retrieved at oocyte pick-up [24].

	Age-Idiopathic Factor *n* = 36	Endometriosis *n* = 23	Male Factor *n* = 11
Total FSH (IU)	2633.33 ± 838.96	2585.87 ± 820.92	2168.18 ± 659.36
Age (years)	38.06 ± 3.13	35.30 ± 3.23 **	33.64 ± 1.21 ***
BMI (kg/m^2^)	22.56 ± 3.2	23.49 ± 3.51	23.43 ± 4.27
AFC	10.36 ± 5.5	9.57 ± 6.06	12.83 ± 4.84
AMH (ng/mL)	1.9 ± 0.71	2.08 ± 1.38	2.43 ± 1.25
FORT	0.75 ± 0.40	0.82 ± 0.67	0.78 ± 0.30
FOI	0.69 ± 0.46	0.64 ± 0.38	0.58 ± 0.31

** *p* < 0.001; *** *p* < 0.0001 comparison vs. Age-Idiopathic Factor group, ANOVA followed by Tukey’s post hoc test.

**Table 2:** IVF outcomes of the three patient groups. Data represent mean ± SD of the parameters evaluated in each group. The fertilization rate was calculated as the number of fecundated oocytes/number of total retrieved oocytes and number of mature oocytes (metaphase II) (MII oocytes). The pregnancy rate was calculated as the number of positive pregnancies/number of the total women with transferred embryos. Comparing patients’ groups, no parameter reached the significance at ANOVA.

Parameters	Patients		
	Age-Idiopathic Factor	Endometriosis	Male Factor
Follicle number	7.25 ± 4.28	7.13 ± 5.03	8.18 ± 4.26
Retrieved oocytes	9.28 ± 5.37	6.87 ± 4.78	10.27 ± 6.02
MII oocytes	7.05 ± 4.61	5.60 ± 4.31	7.45 ± 4.39
Fecundated oocytes	5.16 ± 3.79	3.61 ± 3.94	5.00 ± 3.63
Fertilization rate	0.58 ± 0.29	0.49 ± 0.33	0.48 ± 0.24
Pregnancy (%)	24	11	29

## Data Availability

The original contributions presented in this study are included in the article/Supplementary Materials. Further inquiries can be directed to the corresponding author.

## Supplementary Materials

The following supporting information can be downloaded at https://www.mdpi.com/article/10.3390/biom15040561/s1, Figure S1. Granulosa cells (GCs) differentiation phenotypes; Figure S2. FSHR content in differently treated GCs from AIF, Endo, or MF Group.

## Abbreviations

The following abbreviations are used in this manuscript:

GCs	granulosa cells
FF	follicular fluid
FSHR	follicle-stimulating hormone receptor
Cx43	Connexin-43
LH	Luteinizing Hormone
ART	assisted reproductive technologies
AIF	Age Idiopathic Infertility
ENDO	endometriosis
MF	male factor
ECM	extracellular matrix
HCG	Human Chorioic Gonadotropinn
CM	culture medium
AMH	Anti-Müllerian Hormone
GJ	gap junction
