# Supplementary material for: Endometriotic Follicular Fluid Affects Granulosa Cells’ Morphology and Increases Duplication Rate and Connexin-43 Expression
Source: Biomolecules. 2025 Apr 10;15(4):561. doi: 10.3390/biom15040561 (PMC12024943; doi:10.3390/biom15040561)
Supplement: Supplementary file 1 [file biomolecules-15-00561-s001.zip › biomolecules-3538414-supplementary.pdf]

Supplementary Materials

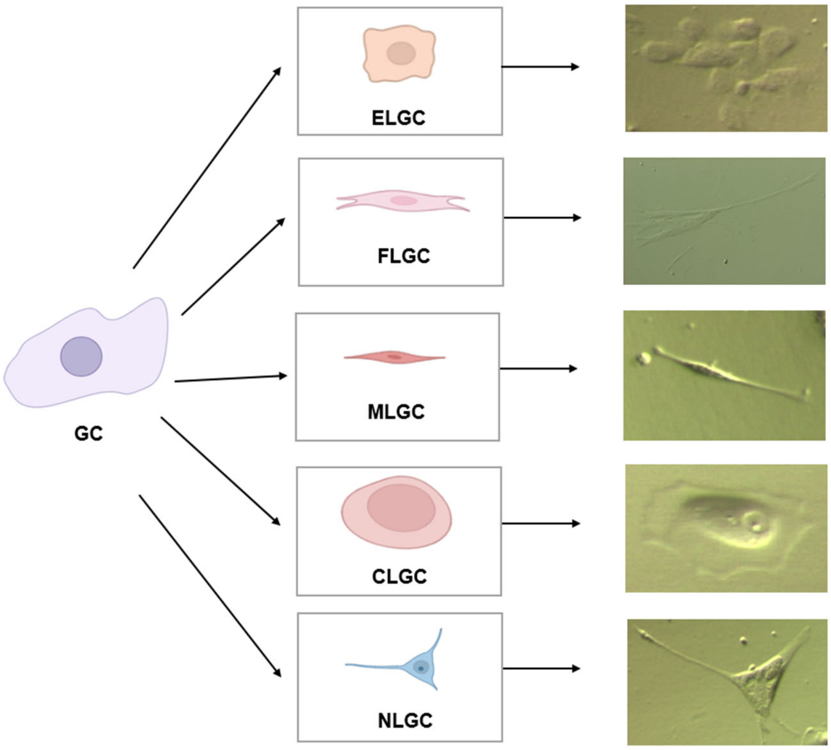

Figure S1. Granulosa cells (GCs) differentiation phenotypes: ELGC epithelial-like granulosa cell; FLGC fibroblast-like granulosa cell; CLGC chondroblast-like granulosa cell; NLGGC neuronal-like granulosa cell.

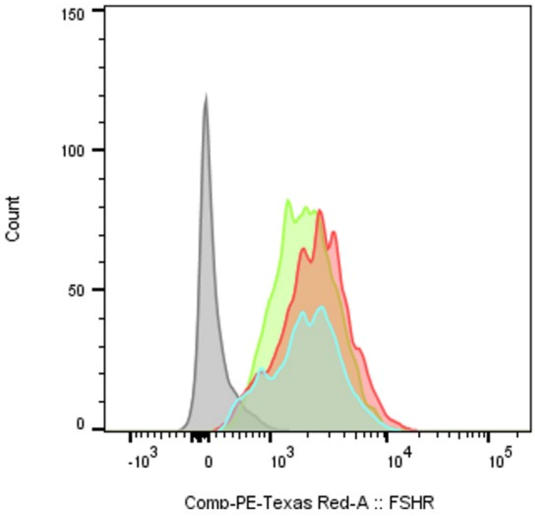

Figure S2. FSHR content in GCs. The expression of FSHR was assessed by flow cytometry in GCs cultured in the presence of FF-AIF (blue histogram), FF-ENDO (red histogram), or FF-MF (green histogram), by comparing the fluorescence shift to the appropriate autofluorescence control (GCs stained with the secondary antibody, grey histogram). These data confirmed the identity of GCs under the three different conditions.
